# Supplementary material for: Density Functional Theory Studies on the Chemical Reactivity of Allyl Mercaptan and Its Derivatives
Source: Molecules. 2024 Jan 31;29(3):668. doi: 10.3390/molecules29030668 (PMC10856204; doi:10.3390/molecules29030668)
Supplement: Supplementary file 1 [file molecules-29-00668-s001.zip › molecules-2828806-supplementary.pdf]

Supporting information

Density Functional Theory Studies on the Chemical Reactivity of Allyl Mercaptan and its Derivatives

Marcin Molski

Quantum Chemistry Department
Faculty of Chemistry
Adam Mickiewicz University
ul. Uniwersytetu Poznańskiego 8, 61-614 Poznań, Poland
mamolski@amu.edu.pl

1. Thermodynamic Descriptors and Scavenging Mechanisms

The free radicals can be deactivated by R─H compound, through the three fundamental mechanisms specified below.

HAT (*Hydrogen Atom Transfer*)

$$R{-}H + X^{\bullet} \rightarrow R^{\bullet} + X{-}H$$

This mechanism is widely applied in processes in which the hydrogen atom is transferred from the basic compound to the reactive intermediate. The antiradical potency of a compound is related to the low bond dissociation enthalpy (BDE) of the N–H bond in amines, S–H in organosulfur compounds, or O–H in polyphenols. Hence, crucial for calculating the BDE parameter is reaction

$$R{-}H \rightarrow R^{\bullet} + H^{\bullet}$$

initiating the HAT process, which allows defining the BDE parameter [20]

$$BDE = H(R^{\bullet}) + H(H^{\bullet}) - H(R - H) \qquad \text{(S1)}$$

in which $H(R^{\bullet})$, $H(H^{\bullet})$ and H (R─H) denote enthalpies of the radical, hydrogen atom, and the neutral molecule.

SET-PT (*Single Electron Transfer Followed by Proton Transfer*)

$$R{-}H + X^{\bullet} \rightarrow R{-}H^{\bullet+} + X^{-}$$

$$R{-}H^{\bullet+} \rightarrow R^{\bullet} + H^{+}$$

This scenario involves the transfer of an electron from the parent compound

$$R{-}H \rightarrow R{-}H^{\bullet+} + e^{-}$$

generating a cation radical $R{-}H^{\bullet+}$ and, in the next step, the proton transfer from the cation radical producing parent agent in the radical form $R^{\bullet}$. These two stages are characterized by the adiabatic ionization potential (AIP) and the proton dissociation enthalpy (PDE) calculated from the formulae [20]

$$\begin{aligned} AIP &= H(R - H^{\bullet+}) + H(e^{-}) - H(R - H) \\ PDE &= H(R^{\bullet}) + H(H^{+}) - H(R - H^{\bullet+}) \end{aligned} \qquad \text{(S2)}$$

Here, $H(R{-}H^{\bullet+})$, $H(e^{-})$, $H(H^{+})$ and H(R─H) represent the enthalpies of the cation, electron, proton, and the parent compound, respectively.

SPLET (*Sequential Proton Loss Electron Transfer*)

$$R\text{—}H \rightarrow R^- + H^+$$

$$R^- + X^\bullet + H^+ \rightarrow R^\bullet + X\text{—}H$$

The second step f the reaction above specified is initiated by the reaction

$$R^- \rightarrow R^\bullet + e^-$$

therefore, the SPLET process is characterized by the proton affinity (PA) and electron transfer enthalpy (ETE) calculated according to the formulae [20]

$$\begin{aligned} PA &= H(R^-) + H(H^+) - H(R-H) \\ ETE &= H(R^\bullet) + H(e^-) - H(R^-) \end{aligned} \qquad \text{(S3)}$$

If specified above reactions are not sequential and involve the concerted shift of a single electron and a single proton they are called Concerted Proton-Electron Transfer (CPET) [39]. This pathway belongs to the wide class of a Proton-Coupled Electron Transfer (PCET) [40], which involves the shift of electrons and protons from one molecule (atom) to another.

TMC (*Transition Metals Chelation*)

This mechanism is related to the chelation of the transition metal ions (especially Fe^{2+} and Cu^{2+}), which produces stable complexes that remove them from the reaction medium and participate in the slowing down the radical cascade reactions. The vital for this mechanism is a dissociation decay

R—H → R- + H+

The parameter characterizing TMC in solvents is the free Gibbs acidity [41]

$$G_{acidity} = G(R^-) - G(R-H) \qquad \text{(S4)}$$

in which $G(R^-)$ and G(R—H) denote the Gibbs free energy of the anion and its parent compound, respectively. A lower value of this parameter indicates a greater ability to chelate metals and, consequently, potency to slow down radical processes.

RAF (*Radical Adduct Formation*)

In this scenario radicals are deactivated in the reaction [42]

$$R\text{—}H + X^\bullet \rightarrow R\text{—}XH^\bullet$$

which is characterized by the enthalpy or Gibbs free energies formation calculated for a given radical and an antioxidant considered [42]. Hence, this pathway is characterized by the descriptor depending on the radical type, consequently, it will not be considered here.

In calculations of the BDE, PDE, AIP, ETE, PA, $G_{acidity}$ parameters, we used the values of the electron, proton and hydrogen enthalpies in the gas phase, water and benzene recommended by Rimarčik et al. [20].

2. Chemical Activity Descriptors

An important source of information on the reactivity of chemical compounds is the difference in the energies of the HOMO and LUMO frontier orbitals. The shape of these orbitals, as well the energy gap (usually expressed in electron volts [eV])

$$\Delta E = E_{LUMO} - E_{HOMO} \tag{S5}$$

have an impact on the chemical reactivity of compounds. A large energy difference defines a *hard* molecule that is more stable and less active, while a small energy gap defines a *soft* molecule that is less stable and more reactive. Based on the energy of the LUMO and HOMO orbitals, as well as the Koopmans' theorem for the closed shell molecules, one may define the global activity descriptors [43,44], which model the physicochemical properties of chemical compounds [45]. The most important chemical activity descriptors are specified below.

Ionization potential

$$IP = -E_{HOMO} \tag{S6}$$

It describes the minimum energy required to remove an electron from the molecule's HOMO orbital and move it to infinity. The small IP value indicates a greater tendency of the molecule to participate in the chemical reaction related to electron transfer. The best radical scavengers are endowed with low IP values.

Electron affinity

$$EA = -E_{LUMO} \tag{S7}$$

It expresses the ability of a molecule to accept an electron and produce an anion. Because radicals scavenging might act either via donating or accepting electrons, the EA descriptor is useful to characterize the capacity of a compound to accept electrons.

Chemical hardness

$$\eta \approx \frac{E_{LUMO} - E_{HOMO}}{2} \tag{S8}$$

It describes a molecule with the low susceptibility to deformation or polarization of the electron cloud under the influence of external factors, e.g., reagents.

Chemical softness

$$S \approx \frac{1}{E_{LUMO} - E_{HOMO}} \tag{S9}$$

The inverse of chemical hardness – the S-descriptor characterizes molecules with the high susceptibility to deformation and polarization of the electron cloud.

Chemical potential

$$\mu \approx \frac{E_{LUMO} + E_{HOMO}}{2} \tag{S10}$$

Employed to describe the thermodynamic activity of substances and used e.g. in the derivation of the phase equilibrium constants.

Electronegativity

$$\chi = -\mu \approx -\frac{E_{LUMO} + E_{HOMO}}{2} = \frac{EA + IP}{2} \tag{S11}$$

It characterizes a tendency to attract electrons that create the chemical bond. The common electron pair is shifted towards the atom having a high value of χ, which is accompanied by the formation of a polar or ionic bond.

Electrophilicity Index

$$\omega \approx \frac{\chi^2}{2\eta} = \frac{\left(E_{LUMO} + E_{HOMO}\right)^2}{4\left(E_{LUMO} - E_{HOMO}\right)} \tag{S12}$$

This descriptor was introduced by Robert Parr and coworkers [46]. It measures the energy change of an electrophilic reagent (acceptor) when it is saturated with electrons provided by a another reagent (donor). The higher value of ω characterizes a strong electrophile, whereas a strong nucleophile is described by lower value of ω.

Electro-donating and electro-accepting power

A radical scavenger (RS) can act in two ways: either by donating electrons to, or accepting electrons from a radical (R)

$$RS + R \rightarrow RS^{+} + R^{-} \qquad RS + R \rightarrow RS^{-} + R^{+}$$

Following this fact, Gázquez et al. [47] introduced the new descriptors characterizing the electro-donating ω^- and electro-accepting ω^+ power of RS, defined in the following manner

$$\omega^{-} = \frac{\left(3IP + EA\right)^2}{16\left(IP - EA\right)} \qquad \omega^{+} = \frac{\left(IP + 3EA\right)^2}{16\left(IP - EA\right)} \tag{S13}$$

To characterize the relative electro-accepting(donating) power of an arbitrary radical scavenger X, Martinez [24] introduced the acceptance Ra and donation Rd indexes

$$Ra = \frac{\omega_X^{+}}{\omega_F^{+}} \qquad Rd = \frac{\omega_X^{-}}{\omega_{Na}^{-}} \tag{S14}$$

defined by the IP and EA for the F and Na atoms. They represent a good electron acceptor (F) and a good donor (Na), respectively. In the calculations, the well-known experimental values [24] for F: IP= 17.42282, EA= 3.4011898 [eV], and for Na: IP= 5.1391, EA= 0.547926 [eV] are employed. The descriptors Ra and Rd are useful for classifying any compound X in terms of its electron donating (accepting) capacity. Hence, Ra and Rd can be applied to characterize the antiradical capacity of X in terms of its electro-donating(accepting) power with respect to the F and Na atoms, taken as the reference points [24].

Figure S1. Optimized geometries of 2-propenesulfenic acid (PSA) and allyl mercaptan (AM) cation (C), radical (R), and anion (A) in water, and AM in water, vacuum and benzene. The enthalpies H(C), H(R) and H(A) [Ha] were calculated using DFT B3LYP/cc-pVQZ level of the theory and C-PCM solvation model.

PSA Water

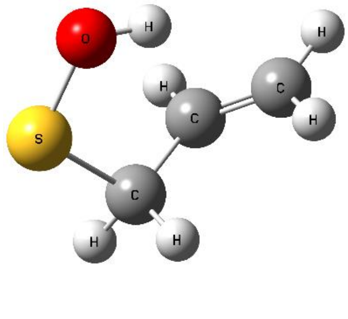

H(C)=-591.123054

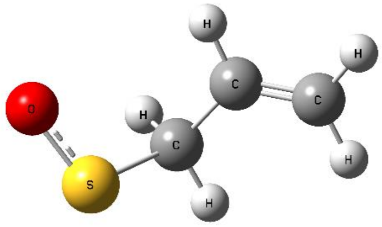

H(R)=-590.735700

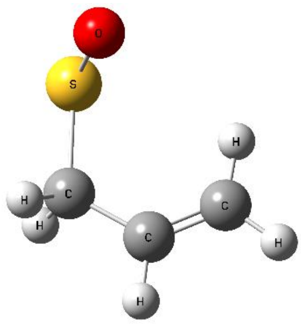

H(A)=-590.863332

AM Water

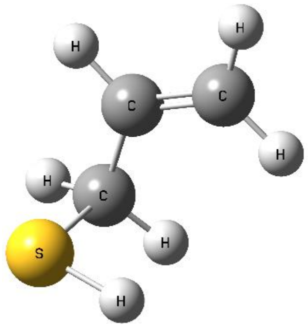

H(C)=-515.871059

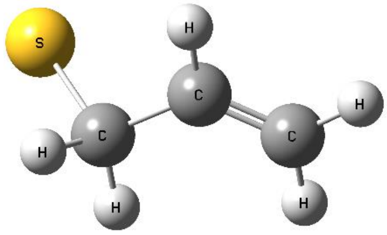

H(R)=-515.473934

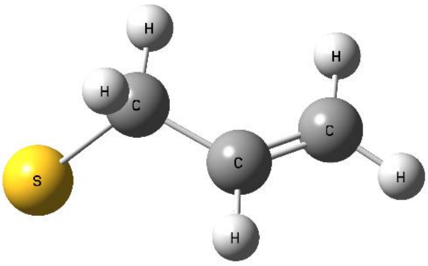

H(A)=-515.642878

AM Vacuum

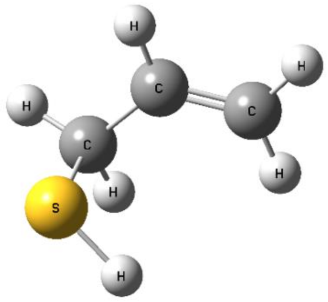

H(C)=-515.784338

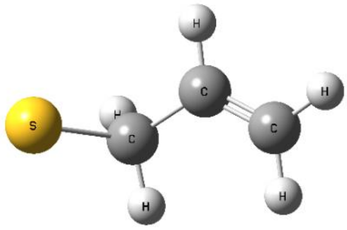

H(R)=-515.470442

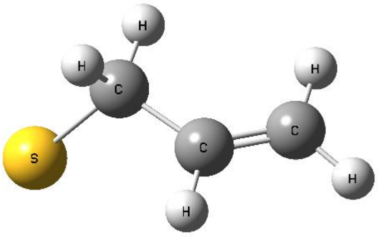

H(A)=-515.542450

AM Benzene

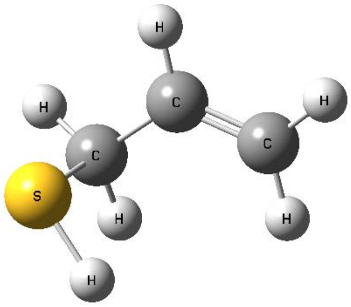

H(C)=-515.833349

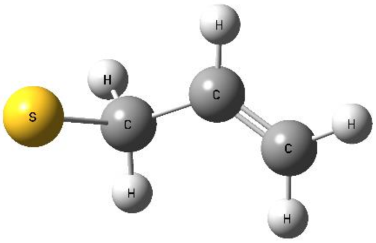

H(R)=-515.472292

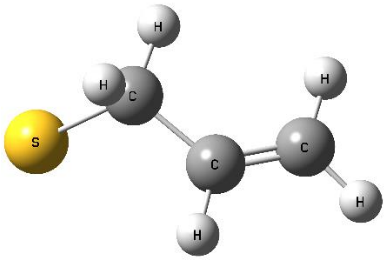

H(A)=-515.597911
